# Use of Recombinant Adenovirus Vectored Consensus IFN-α to Avert Severe Arenavirus Infection

**DOI:** 10.1371/journal.pone.0026072

**Published:** 2011-10-24

**Authors:** Brian B. Gowen, Jane Ennis, Andrew Russell, Eric J. Sefing, Min-Hui Wong, Jeffrey Turner

**Affiliations:** 1 Institute for Antiviral Research and Department of Animal, Dairy, and Veterinary Sciences, Utah State University, Logan, Utah, United States of America; 2 Defyrus Inc., Toronto, Ontario, Canada; Veterinary Laboratories Agency, United Kingdom

## Abstract

Several arenaviruses can cause viral hemorrhagic fever, a severe disease with case-fatality rates in hospitalized individuals ranging from 15-30%. Because of limited prophylaxis and treatment options, new medical countermeasures are needed for these viruses classified by the National Institutes of Allergy and Infectious Diseases (NIAID) as top priority biodefense Category A pathogens. Recombinant consensus interferon alpha (cIFN-α) is a licensed protein with broad clinical appeal. However, while cIFN-α has great therapeutic value, its utility for biodefense applications is hindered by its short *in vivo* half-life, mode and frequency of administration, and costly production. To address these limitations, we describe the use of DEF201, a replication-deficient adenovirus vector that drives the expression of cIFN-α, for pre- and post-exposure prophylaxis of acute arenaviral infection modeled in hamsters. Intranasal administration of DEF201 24 h prior to challenge with Pichindé virus (PICV) was highly effective at protecting animals from mortality and preventing viral replication and liver-associated disease. A significant protective effect was still observed with a single dosing of DEF201 given two weeks prior to PICV challenge. DEF201 was also efficacious when administered as a treatment 24 to 48 h post-virus exposure. The protective effect of DEF201 was largely attributed to the expression of cIFN-α, as dosing with a control empty vector adenovirus did not protect hamsters from lethal PICV challenge. Effective countermeasures that are highly stable, easily administered, and elicit long lasting protective immunity are much needed for arena and other viral infections. The DEF201 technology has the potential to address all of these issues and may serve as a broad-spectrum antiviral to enhance host defense against a number of viral pathogens.

## Introduction

The *Arenaviridae* family of viruses has several members that can cause viral hemorrhagic fever, an acute, often-fatal, viral syndrome characterized by intense fever, malaise, and less frequently, bleeding and neurologic manifestations. Case fatality rates of hospitalized patients suffering from arenaviral hemorrhagic fever (AHF) range from 15–30% [1], [2], [3], [4]. Arenaviruses known to cause AHF include Junín, Machupo, Guanarito, Sabiá, and Chapare in the South American continent, and Lassa and Lujo in west and southern Africa, respectively. Primary transmission of the arenaviruses from respective rodent reservoir hosts to humans occurs via exposure to contaminated excreta [5]. Person-to-person transmission can occur through contact with blood or other body fluids during the care and management of infected individuals [1], [6]. Notably, these viruses are considered a threat to national security and are classified as highest priority pathogens by the NIAID [7].

At present, the treatment of AHF is limited to ribavirin and immune plasma [8], [9]. The latter has only been proven to be effective in treating cases of Argentine hemorrhagic fever (Junín virus infection) within 8 days of disease onset. Off-label usage of ribavirin has been shown to be effective in treating Lassa fever when therapy was initiated within 6 days of the development of clinical symptoms. However, there are toxicities associated with ribavirin therapy at dosages required for efficacious use, which may contribute to the observed poor patient compliance in completing prescribed treatment regimens [10], [11]. Very limited case data using ribavirin to treat other AHFs supports the use of emergency protocols [1], [12], [13], however the utility of ribavirin therapy remains to be seen.

Interferon alpha (IFN-α) is an effective part of the host innate immune response, which can be manufactured as a recombinant human protein with broad clinical appeal [14]. Consensus (c)IFN-α, also known as IFN alfacon-1 and Infergen, is a licensed, second generation IFN-α engineered to contain the most frequently occurring amino acids among the nonallelic IFN-α subtypes. Previously, we have demonstrated that cIFN-α can be used effectively alone, or in combination with ribavirin, to treat Pichindé virus (PICV) infection in hamsters [15], [16], an experimental model of acute arenaviral disease [17]. However, while cIFN-α has clinical value, its usefulness is hindered by its short half-life and cost to manufacture. There is an initial distributive half-life of 7 minutes and a beta half-life of 2 to 5 hours [14]. The rapid systemic clearance requires frequent dosing to achieve desired therapeutic levels. Consequently, treatment can result in well-documented toxicities which include headache, depression, hair loss, fever, and malaise. In order to combat the rapid degradation, PEGylated forms of recombinant IFN-α have been introduced with half-lives that are on the order of days instead of hours, thus reducing the number of injections to once per week [18]. However, the cost to manufacture PEG-IFN-α is exceedingly high, and the PEGylation process has been shown to reduce the activity of IFN-α, thereby further increasing the production costs.

To circumvent the fast decay of cIFN-α, a replication-incompetent, recombinant adenovirus type 5 (rAd5) gene delivery platform was designed to drive constitutive expression of the cIFN-α gene from transduced nasal epithelial target cells. This rAd5 cIFN-α virus, called DEF201, was first developed in mice and recently shown to be active against yellow fever virus (YFV) infection in hamsters [19], [20]. The intranasal (i.n.) inoculation used in the YFV study prevents the host immune system from recognizing the Ad5 vector, thereby bypassing any possible pre-existing immunity [21]. In the present study, we evaluated the use of DEF201 administered i.n. for the prevention and treatment of PICV infection in hamsters.

## Materials and Methods

### Ethics statement

All animal procedures complied with USDA guidelines and were conducted at the AAALAC-accredited Laboratory Animal Research Center at Utah State University under protocol 1229, approved by the Utah State University Institutional Animal Care and Use Committee.

### Animals

Female golden Syrian hamsters were obtained from Charles River Laboratories (Wilmington, MA) and acclimated for a minimum of 6 days prior to experimentation. They were fed standard hamster chow and tap water *ad libitum*. Animals were approximately 7–9 weeks old at the time of virus challenge.

### Viruses

PICV, strain An 4763, was provided by Dr. David Gangemi (Clemson University, Clemson, South Carolina). The virus was passaged once through hamsters. Virus stocks were prepared from pooled livers harvested from infected hamsters. Virus dilutions were made in minimal essential medium (MEM), and infectious inoculum was given bilaterally in two intraperitoneal (i.p.) injections of 0.1 mL each. The recombinant adenovirus vectored cIFN-α (rAd5-huIFN-α; DEF201) and the rAd5 empty vector (rAd EV) control virus were provided by Defyrus, Inc. (Toronto, ON, Canada) at a concentration of 6×10^9^ and 2×10^11^ plaque-forming units (pfu)/ml, respectively. Both viruses were prepared in PBS for i.n. instillation in a 200 µl volume.

### Liver, spleen and serum virus titers

Virus titers were assayed using an infectious cell culture assay as previously described [22]. Briefly, a specific volume of liver or spleen homogenate or serum was serially diluted and added to triplicate wells of Vero (African green monkey kidney; American Type Culture Collection, Manassas, VA) cell monolayers in 96-well microplates. The viral cytopathic effect (CPE) was determined 7 to 8 days post-virus inoculation, and the 50% endpoints were calculated as described [23]. The assay detection ranges were 2.8 to 9.5 log_10_ 50% cell culture infectious doses (CCID_50_)/g of liver or spleen and 1.8 to 8.5 log_10_ CCID_50_/ml of serum. In samples presenting with undetectable liver or spleen virus, a value of <2.8 was assigned (<1.8 for serum). Conversely, in cases wherein virus exceeded the detection range, a value of>9.5 (>8.5 for serum) was assigned. For statistical analysis, values of 2.8 or 9.5 log_10_ (1.8 or 8.5 for serum) were assigned as needed for samples with undetectable or saturated virus levels, respectively.

### Serum alanine aminotransferase (ALT) determinations

Detection of ALT in serum is an indirect method for evaluating liver disease. Serum ALT levels were measured using the ALT (SGPT) Reagent Set purchased from Pointe Scientific, Inc. (Lincoln Park, MI) per the manufacturer's recommendations. The reagent volumes were adjusted for analysis on 96-well microplates.

### Experimental design

#### DEF201 dose range titration experiment

Hamsters were weighed on the morning prior to the day of infection and grouped (n = 15 for drug treatment groups, 26 for the placebo group) so that the average hamster weight per group across the entire experiment varied by less than 5 grams. Varying pfu amounts of DEF201, the rAd EV control virus, or saline placebo treatments were administered in a single i.n. dose 24 h prior to challenge with ∼5 pfu of PICV. Five animals from each group were sacrificed on day 7 of infection. Serum was collected for assaying ALT activity, and virus titers were determined for liver, spleen, and serum samples as described above. The remaining 10 animals (21 for the placebo group) were observed 21 days for mortality and weighed individually every 3 days starting on day 0. Sham-infected normal controls (n = 3) were included for comparison.

#### Extended pre-exposure prophylaxis experiment

The design was similar to the DEF201 titration experiment with the following differences. Hamsters were weighed on the morning of initial pretreatment (day −14 relative to the infection) and grouped (n = 15 per group). Groups were treated once i.n. with 10^8^ pfu of DEF201, rAd EV control virus, or saline placebo. Treatments were given 14 or 7 days prior to challenge with ∼5 PFU of PICV. Animals were observed for 28 days post-challenge for mortality.

#### Post-exposure prophylaxis experiment

The design was similar to the DEF201 pre-exposure prophylaxis experiment with the following differences. Single dose i.n. treatments with 10^8^ pfu of DEF201 or rAd EV were administered 24 h prior to, or 6, 24, or 48 h after challenge with ∼5 pfu of PICV. On day 28 post-infection, the surviving animals (including 6 naïve sham-infected controls) were re-challenged. Morbidity and mortality were observed out to 58 days after the initial challenge.

### Statistical analysis

Kaplan-Meier survival plots and all statistical evaluations were done using Prism (GraphPad Software, CA). The log-rank test was used for survival analysis. For analyzing differences in viral titers, ALT levels, and weight change, a one-way analysis of variance (ANOVA) with Newman-Keuls post test or the Kruskal-Wallis (two-tailed) test with the Dunn's post test was performed based on Gaussian distribution of the data.

## Results

### DEF201 protects hamsters from lethal PICV challenge

**Figure 1:**
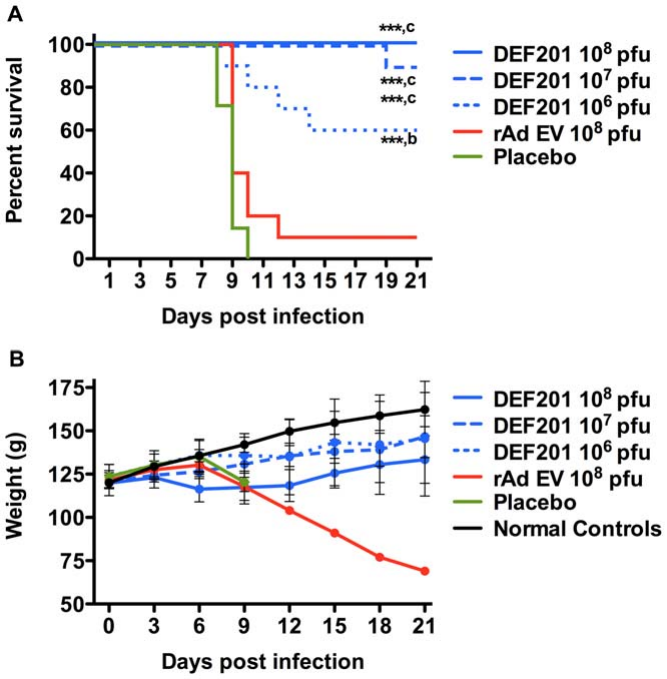
DEF201 pretreatment protects PICV-infected hamsters from mortality. Animals were treated 24 hours prior to infection with a single i.n. instillation of the indicated dose of DEF201, the rAd EV control virus, or PBS placebo. (A) Survivial and (B) average weights and standard deviations of surviving animals (measured every 3^rd^ day) are shown. ***P<0.001 compared to placebo-treated animals. ^b^ *P*<0.01 and ^c^ *P*<0.001 compared to rAd EV-treated animals.

In the initial trial, hamsters were treated with 10^6^ to 10^8^ pfu of DEF201 one day prior to challenge with a lethal dose of PICV. Pretreatment with the highest dose of 10^8^ pfu of DEF201 resulted in 100% survival, and 10^7^ and 10^6^ pfu doses also significantly protected 90% and 60% of hamsters, respectively, from mortality (Figure 1A). Moreover, the hamster that succumbed in the 10^7^ group, survived 19 days. Importantly, only one out of ten hamsters treated with 10^8^ pfu of the control rAd EV virus survived the infection; however, there did appear to be a slight delay in the time to death in the hamsters that received the control virus treatment.

The weights of the hamsters were measured every 3 days to assess weight gain over the course of the experiment as a marker of well being (Figure 1B). Notably, from day 3 to day 6, a time before weight loss due to illness from PICV infection would have been expected, hamster weights decreased as the dose of DEF201 increased. This would suggest that the higher treatment doses may have resulted in some loss of appetite, probably due to mild illness due to expression of consensus IFN since no overt effects were noticeable when handling the animals. The hamsters that received the 10^6^ pfu dose of DEF201 gained weight through day 6 similarly to the animals treated with saline placebo and the normal controls (sham-infected, untreated) (Figure 1B). The high-dose of rAd EV control virus also resulted in a slight reduction in weight compared to the controls, suggesting that the immune response to the adenoviral vector alone may have caused some malaise in the animals.

**Figure 2:**
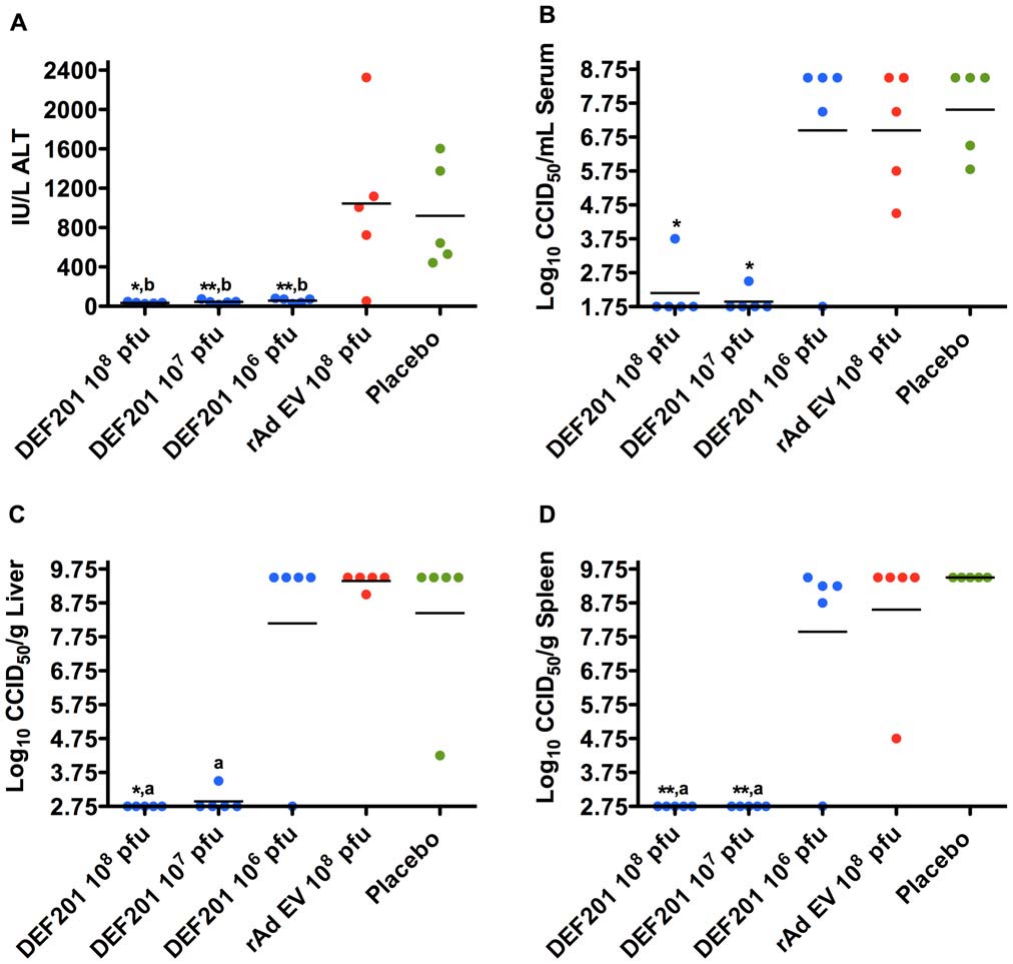
DEF201 prophylaxis limits liver disease and viral loads in PICV-infected hamsters. Animals were treated as described in Figure 1 and sacrificed on day 7 of infection for analysis of serum (A) ALT and (B) virus titer, and (C) liver and (D) spleen viral titers. *P<0.05, **P<0.01, compared to placebo-treated animals. ^a^ *P*<0.05, ^b^ *P*<0.01, compared to rAd EV-treated animals.

There was no elevation in serum ALT levels on day 7 of infection in samples collected from parallel treated and infected hamsters receiving DEF201 (Figure 2A). Eighty percent of the rAd EV group and 100% of the PBS placebo group had elevated levels of ALT, reflective of liver disease. Interestingly, the 10^7^ and 10^8^ pfu DEF201 groups presented with little to no day-7 virus burden in the serum, liver, or spleen, while the 10^6^ group developed viral titers that were comparable to the rAd EV and placebo controls (Figure 2B-D). A delay in the development of liver disease in the 10^6^ pfu DEF201-treated animals may explain the reduced ALT levels. Alternatively, saturation of liver virus titers in the low-dose DEF201, rAd EV, and placebo groups may have masked a substantial difference between the former and the viral vector and vehicle control groups.

### DEF201 extended prophylaxis against PICV infection

**Figure 3:**
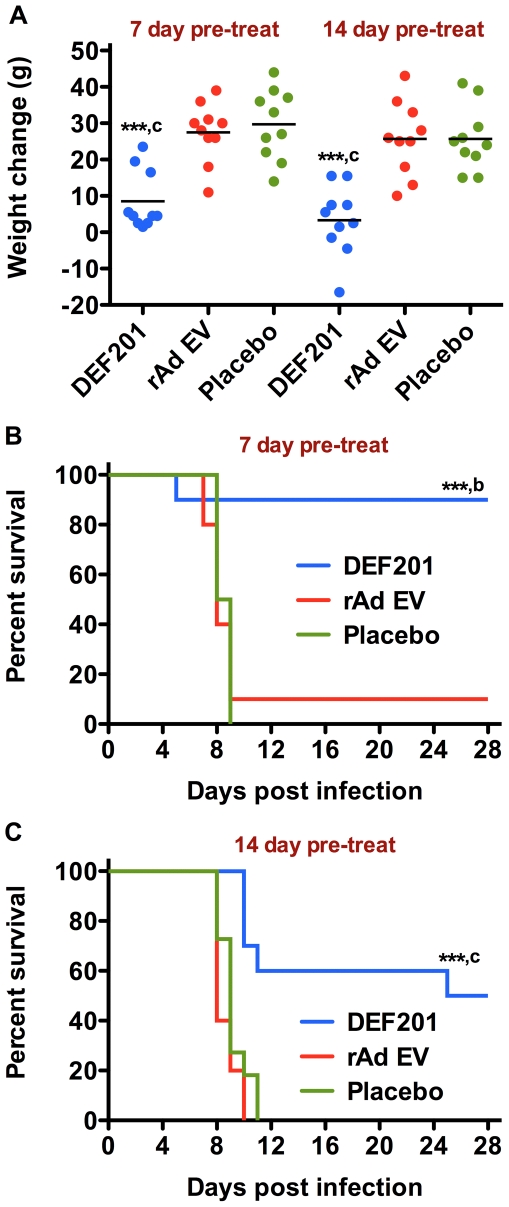
DEF201 extended pre-exposure prophylaxis protects hamsters from lethal PICV challenge. Animals were treated i.n. with a single dose 10^8^ pfu of DEF201, the rAd EV control virus, or PBS placebo 7 or 14 days prior to PICV infection. Animal weights were measured two weeks prior to, and at the time of, PICV challenge. The effect of 7-day and 14-day pretreatments on A) weight change over the two-week period prior to PICV challenge and the extended PICV prophylaxis efficacy data for the B) 7-day and C) 14-day pretreatments are shown. ***P<0.001 compared to respective placebo-treated animals. ^b^ *P*<0.01, ^c^ *P*<0.001 compared to respective rAd EV-treated animals.

We next evaluated the prophylactic window of protection against PICV infection using the 10^8^ pfu dose of DEF201. Animals were treated one or two weeks prior to challenge with a lethal dose of PICV. Consistent with the trend observed in initial dose titration study, hamsters treated with the 10^8^ pfu dose of DEF201 had significantly reduced weights compared to those that received the rAd EV and placebo control treatments (Figure 3A). Nevertheless, the pretreatment with DEF201 seven days before infection was highly protective (90% survival rate; Figure 3B). Notably, the single hamster that failed to survive the challenge succumbed on day 5, which was several days before the mean time to death measured in both the placebo and rAd EV groups. An autopsy to determine the cause of death was not performed.

In hamsters treated two-weeks prior to PICV challenge, DEF201 significantly reduced mortality (50% survival) and extended the time of death in the animals that succumbed (Figure 3C). In contrast, uniform lethality was seen with animals that received the rAd EV and placebo treatments. Of the 5 surviving animals pre-treated with DEF201, one was anorexic at the conclusion of the study on day 28 post-infection. This was reflected by a 27% weight loss compared to the animals starting weight. It is possible that this hamster, which appeared ill and lethargic, was not able to completely prevent the infection. It was unclear whether it would have ultimately recovered if the observation period had been extended.

**Figure 4:**
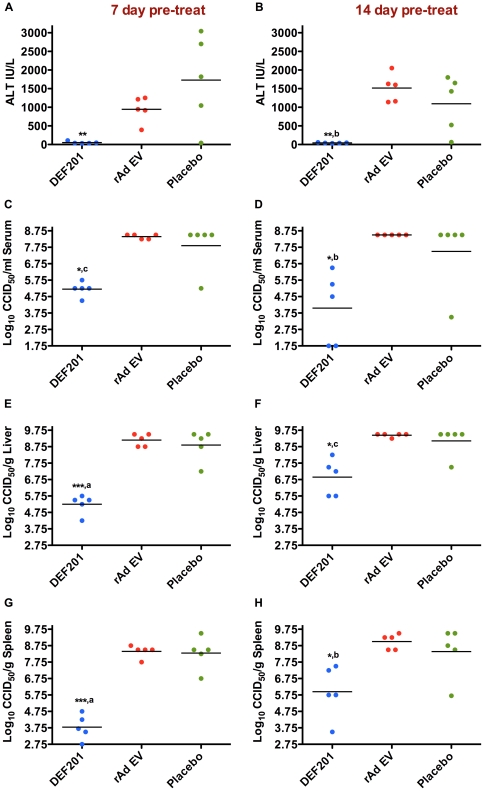
DEF201 extended pre-exposure prophylaxis limits liver disease and viral loads in PICV-infected hamsters. Animals were treated as described in Figure 3, 7 days (A, C, E, G) or 14 days (B, D, F, H) prior to PICV challenge, and sacrificed on day 7 of infection for analysis of serum (A, B) ALT and (C, D) virus titers, and (E, F) liver and (G, H) spleen viral titers. *P<0.05, **P<0.01, ***P<0.001 compared to placebo-treated animals. ^a^ *P*<0.05, ^b^ *P*<0.01, ^c^ *P*<0.001 compared to rAd EV-treated animals.

On both the 7-day (Figure 4A, C, E, G) and 14-day (Figure 4B, D, F, H) pretreatments, DEF201 significantly reduced day-7 viral loads and liver disease (ALT) compared to the controls. The absence of elevated ALT levels in the DEF201-treated hamsters may be explained by the 2–3 log_10_ reduction in liver virus burden (Figure 4E, F) and a delay in the development of liver disease. Although tissue titers were slightly lower when DEF201 was given 7 days prior to challenge compared to the 14 day pretreatment, this was not evident with serum viral burden. Because most animals had measurable replicating PICV (Figure 4C–H), it is likely that survivors would have been immunized and protected from subsequent challenge. This may not be the case with hamsters treated with DEF201 24 h prior to challenge since most had no detectable virus titers in spleen, liver, or serum on day 7 of PICV infection (Figure 2B–D).

### DEF201 post-exposure prophylaxis and acquired immunity against PICV re-challenge

**Figure 5:**
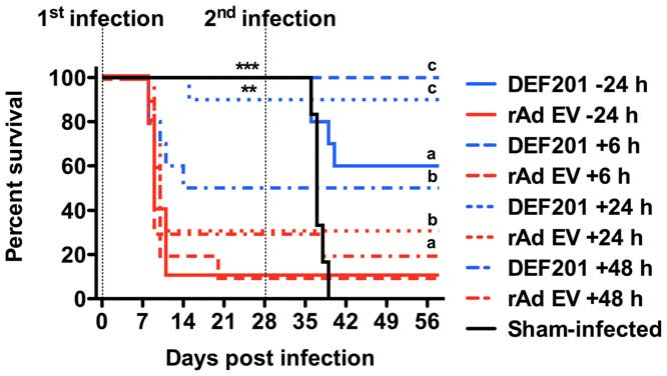
Early DEF201 post-exposure-treatment protects PICV-infected hamsters from mortality. Animals were treated with a single i.n. dose of 10^8^ pfu of DEF201 or the rAd EV control 24 h prior to infection, or 6, 24, or 48 h post infection. The dashed lines represent the 1^st^ and 2^nd^ challenges of the animals with PICV on day 0 and 28 of the experiment, respectively. For the initial infection, **P<0.01, ***P<0.001 compared to respective rAd EV-treated controls. For the second infection, sham-infected animals from the 1^st^ infection were challenged with PICV and compared to groups of surviving animals; ^a^ *P*<0.05, ^b^ *P*<0.01, ^c^ *P*<0.001.

Having observed dramatic protection when administered up to 2 weeks prior to challenge, a final experiment was conducted to determine the therapeutic value of DEF201 in the hamster PICV infection model. When DEF201 was administered 6 or 24 h after challenge, highly significant protection was observed (Figure 5). Efficacy waned when DEF201 treatment was delayed to 48 h post-infection. As anticipated, the treatment given 24 pre-challenge verified previous activity, with all animals surviving challenge. Interestingly, there was higher than expected survival with the control rAd EV treatments initiated 24 and 48 h post-challenge, suggestive of a slight antiviral effect as the time of treatment was further delayed (Figure 5).

The surviving hamsters from this experiment were re-challenged with PICV to assess the ability of DEF201 to enhance longer-term protection via acquired immunity. With the exception of 4 animals in the 24 h DEF201 pretreatment group, and a single animal in the rAd EV 48 h group, all animals that were challenged with PICV on day 0 of the experiment survived a second challenge on day 28 (Figure 5). All six naïve animals that were initially sham-infected succumbed as expected.

**Figure 6:**
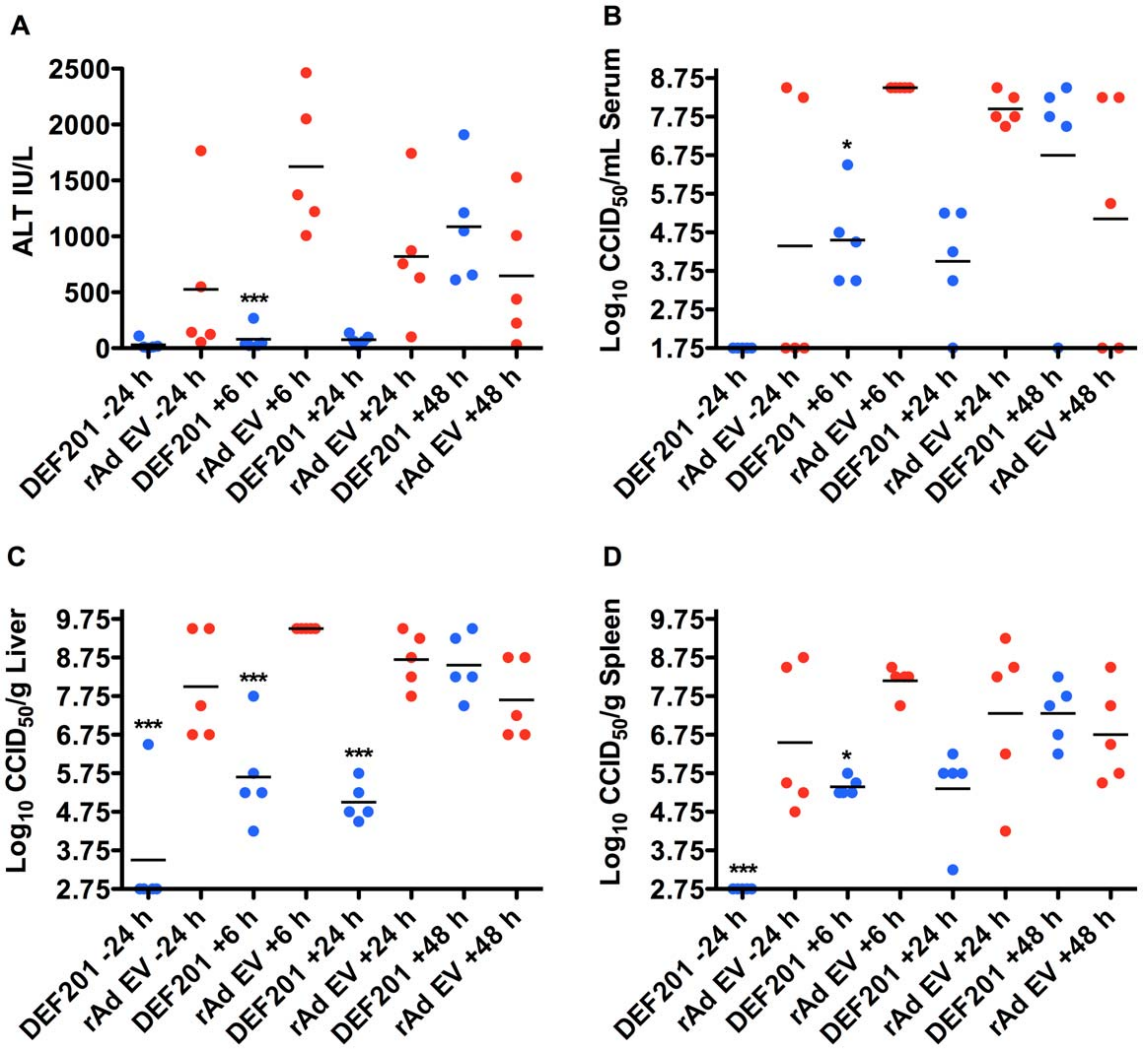
Early DEF201 post-exposure treatment limits liver disease and viral loads in PICV-infected hamsters. Animals were treated as described in Figure 5 and sacrificed on day 7 of infection for analysis of serum (A) ALT and (B) virus titer, and (C) liver and (D) spleen viral titers. *P<0.05, ***P<0.001 compared to rAd EV-treated animals.

In animals that were sacrificed on day 7 relative to the first infection, reductions in ALT and viral titers were most evident in the groups that received DEF201 within 24 h of infection (Figure 6). Notably, in the animals treated with the control rAd EV, there was an interesting trend that developed with the 6 h post-infection group having the greatest ALT levels and viral titers, followed by the 24, 48, and −24 h groups. This trend may suggest a low-level immune stimulation in the hamsters relative to the time at which the rAd EV was given. The resulting lack of measurable viral replication in the −24 h DEF201 group (Figure 6B–D) is likely insufficient to elicit immunological memory. It is unclear as to why one of the first infection survivors from the 48 h rAd EV group ultimately succumbed to the second infection.

## Discussion

In the present study, our findings demonstrate that expression of cIFN-α following a single i.n. administration of DEF201 offers a strong protective effect in hamsters against challenge with PICV that included limiting liver disease and inducing an antiviral state that inhibited systemic and tissue viral replication. The lack of significant antiviral activity elicited by the rAd EV control virus suggests that the enhanced antiviral response produced by DEF201 is largely due to the expression of the cIFN-α gene. The weak stimulatory effect seen in 1 of the 3 experiments was not surprising considering the number of host systems that play a role in sensing the adenovirus vector [24]; however, the effect was short-lived. In contrast, the enhancement of the host antiviral defenses by DEF201 was long-lasting with a 14-day pre-PICV challenge prophylactic window. Moreover, DEF201 was effective when given 1–2 days post-PICV infection. These data also suggest that sufficient viral replication may be necessary to elicit an adaptive immune response that confers lasting protective immunity, as, for the most part, only re-challenged animals from the 24 h DEF201 pretreatment group succumbed to a second challenge with a lethal PICV inoculum. Presumably, the robust innate immunity and antiviral state induced by the DEF201 pretreatment rapidly controlled the ∼5 pfu challenge dose obviating the development of the adaptive immune response and immunological memory.

The pathogenic arenaviruses have evolved strategies to suppress and evade the host immune response [25], [26], [27], [28], resulting in uncontrolled replication and broad dissemination. However, they appear to be unable to block the induction of IFN stimulated genes via exogenous type I IFN [29], which may, in part, explain the success of DEF201 and cIFN-α treatments [15]. Also essential to the success of DEF201 was early intervention prior to significant viral replication and engagement of innate immune suppressive functions. Indeed, early induction of a strong type I IFN response is associated with favorable disease outcome in nonhuman primates challenged with Lassa virus [30]. Early post-exposure prophylaxis was also required with exogenous cIFN-α protein administered by the i.p. route [15], [16]. With the multiple strategies that arena and other pathogenic viruses have in place to subdue the IFN-mediated host antiviral response [31], the utility of DEF201, recombinant IFN proteins, and IFN inducing agents will depend upon the nature of the IFN pathway blockade and require early administration to be effective post-exposure.

Notably, with daily cIFN-α protein injections of up to 40 µg/kg, significant protection was observed; however, survival rates did not exceed 80% in those studies employing the same PICV hamster model system and virus stock [15], [16]. In contrast, DEF201 consistently elicited greater protection (90–100%). The improved efficacy observed with DEF201 may be explained by a combination of factors that includes constitutive expression of fully glycosylated protein and reduced animal stress levels by avoiding daily injections for 7–10 days. We hypothesize that with the appropriate dose of DEF201, therapeutic levels of consensus IFN-α can be maintained, effectively eliminating the daily bolus effect produced by i.p. injections. In addition, because cIFN-α is produced in genetically engineered *Escherichia coli*, the native glycosylation pattern is lost. Conceivably, enhanced immunotherapeutic activity results from fully glycosylated cIFN-α expressed from cells transduced with DEF201.

Previous studies in mice with a related DEF201 virus expressing mouse IFN-α (mDEF201) have shown the utility of adenovirus-based system to counter viral infections [20], [32], [33]. More recently, in a different hamster model of viral hemorrhagic fever, several of us reported on efficacy of DEF201 in mitigating YFV infection and disease [19]. YFV infection appears to be more sensitive to the effects of DEF201, as a lower dose was able to provide complete protection. Taken together with the results of the present study, the experimental animal data support the broad use of DEF201 for extended pre-exposure and early post-exposure prophylaxis applications. Further investigations using advanced arenavirus models based on challenge of nonhuman primates with pathogenic arenaviruses [17] are needed to better evaluate the potential of DEF201 to prevent severe disease in humans. Nonhuman primate models should allow the full spectrum of cIFN-α activity not possible in hamsters or guinea pigs.

The familiarity of the FDA with adenovirus gene delivery technology and approved cIFN-α protein support the development of DEF201 for clinical use. An important step in the development process is the safety/toxicology testing in rodents, which is presently underway. In our studies, the highest dose of 10^8^ pfu of DEF201 administered by the i.n. route appeared to be well-tolerated in hamsters despite evidence of weight loss. They did not appear visibly ill, but clearly the treatment was having some effect that possibly led to reduced food and water consumption consistent with mild toxicity seen with IFN-α therapy. The i.n. delivery route is designed to circumvent pre-existing immunity to adenovirus type 5 in humans [21], [34], and may limit systemic inflammation that could occur by parenteral administration of large numbers of adenovirus particles. Ultimately, the production of a shelf-stable, powdered formulation of DEF201 for easy i.n. administration and long-term storage would be ideal for stock-piling in the event of the need for mass distribution due to intentional release or (re)emerging disease outbreaks of arena or other viral etiology.
